# Familial clustering of Leiomyomatosis peritonealis disseminata: an unknown genetic syndrome?

**DOI:** 10.1186/1471-230X-5-33

**Published:** 2005-10-13

**Authors:** Niels Halama, Silke A Grauling-Halama, Isam Daboul

**Affiliations:** 1 Division of Gastroenterology, Department of Internal Medicine, Medical College of Ohio, 3000 Arlington Avenue, Toledo, Ohio 43614, USA

## Abstract

**Background:**

Leiomyomatosis peritonealis disseminata (LPD) is defined as the occurrence of multiple tumorous intraabdominal lesions, which are myomatous nodules. LPD is a rare disease with only about 100 cases reported. The usual course of LPD is benign with the majority of the patients being premenopausal females. Only two cases involving men have been reported, no syndrome or familial occurrence of LPD has been described.

**Case presentation:**

We describe a Caucasian-American family in which six members (three men) are diagnosed with Leiomyomatosis peritonealis disseminata (LPD) and three deceased family members most likely had LPD (based on the autopsy reports). Furthermore we describe the association of LPD with Raynaud's syndrome and Prurigo nodularis.

**Conclusion:**

Familial clustering of Leiomyomatosis peritonealis disseminata (LPD) has not been reported so far. The etiology of LPD is unknown and no mode of inheritance is known. We discuss possible modes of inheritance in the presented case, taking into account the possibility of a genetic syndrome. Given the similarity to other genetic syndromes with leiomyomatosis and skin alterations, we describe possible similar genetic pathomechanisms.

## Background

**Figure 1:**
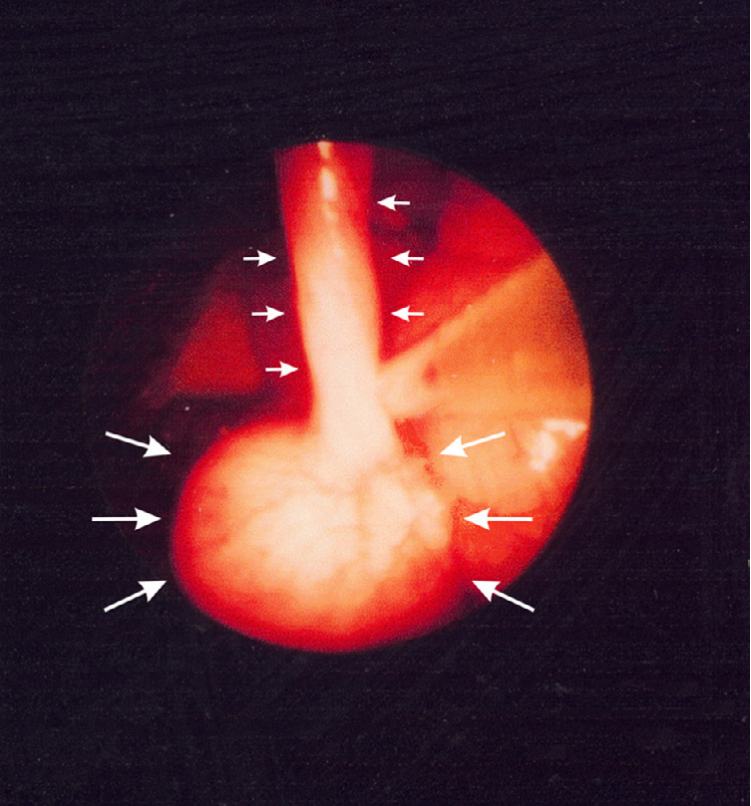
**Intraabdominal picture of a leiomyoma. **Intraabdominal picture taken on endoscopic laparoscopy, showing a pedunculated tumor on the bowels (large white arrows indicate the "head" and small white arrows indicate the "trunk" of the leiomyoma).

**Figure 2:**
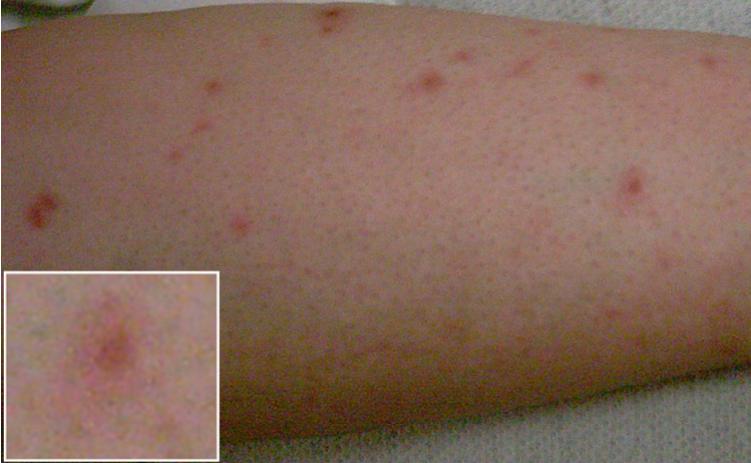
**Skin lesions. **Right lower extremity with multiple itching nodules, excoriations from scratching and general lichenification of the skin (inset: enlarged lesion with excoriation and reddish halo).

Leiomyomatosis peritonealis disseminata (LPD) is a rare smooth muscle tumor, which is characterized by multiple subperitoneal or peritoneal nodules. These myomatous nodules vary in size and can be found on any omental or peritoneal surface of the abdomen, often additional leiomyoma of the uterus are present. No clear correlation between LPD and uterine leiomyomas could be found so far, cases with and without uterine leiomyomas have been reported. Typical histological features are fusiform smooth muscle cells in interdigitating fascicles with no or low-grade cell atypia or mitotic figures [1,2]. Some studies have shown additional features like marked vascularity, without evidence of vascular invasion [3]. Ultrastructural investigations demonstrated abundant intracellular contractile fibers, a basement membrane surrounding every cell, mitochondria at the nuclear poles, stretched oval-shaped nucleoli with an uniform pattern and multiple pinocytic vesicles on the cell surface [4,5]. These findings resemble a developmental stage between fibroblast and mature smooth muscle cell. Immuno-histochemical testing revealed positive results for SMA (smooth muscle antigen), vimentin and desmin [6].

Incidental detection of these abdominal nodules during diagnostic imaging studies occurs frequently and most of the patients are asymptomatic. Symptoms -if any- are often abdominal masses, distension or increasing abdominal girth. Most of the patients present to the gastroenterologist or the gynecologist for further evaluation. Only one case showed an acute abdomen due to acute bowel obstruction as presenting symptom [7].

Differential diagnosis of carcinomatosis peritonealis or abdominal disseminated malignancy presents a major diagnostic problem and only biopsy and histological analysis can reveal the underlying disease. Only two cases of males with LPD have been reported, the majority of patients with LPD are premenopausal woman with high-estrogen states (e.g. pregnancy, oral contraceptive use or the presence of an estrogen-producing neoplasm). The underlying mechanism of LPD development is not known, but involvement of estrogens as etiologic factors has been postulated. Animal studies showed that prolonged elevated levels of estrogen can cause metaplasia of mesenchymal stem cells into fibroblasts, leiomyocytes or endometrial stroma [8]. These animals developed disseminated leiomyomatous peritoneal lesions, similar to LPD. High levels of estrogen and progesterone receptors are found on LPD cells, suggesting potential hormonal responsiveness of these tumors. Reducing excess estrogen levels, removal of estrogen exposure or chemical castration with gonadotropin-releasing hormone agonists can result in regression of the tumor masses [9], cases with successful surgical removal of nodules have also been reported. No definitive therapeutical concept has evolved so far. Furthermore two histological confirmed LPD cases with estrogen- and progesteron-receptor negative tumors have been reported, one in an elderly man and one in a 27 year old woman. The number of LPD cases reported so far is approximately 100, the first case being reported in 1952 by Willson and Peale. So far no familial clustering or involvement of LPD in any syndrome has been found, however genetic syndromes like leiomyomatosis of the esophagus (X-linked Alport syndrome, [10]) or genitourinary tract (multiple cutaneous and uterine leiomyomatosis, MCUL, [11]) are well known.

In this manuscript we describe a Caucasian-American family with several individuals of different generations affected by LPD, half of them being males. Furthermore the additional presence of two clinical traits (Raynaud's syndrome and Prurigo nodularis) was found in all LPD-patients, from whom precise clinical data was obtainable. In addition, we have conducted a review of the literature specifically involving LPD and similar syndromes to evaluate the modes of inheritance. We review these findings and comment on the possible molecular mechanisms leading to the occurrence of LPD (or a genetic syndrome involving LPD) in this family.

## Case presentation

The propositus in this family (IV6) is a 45 year old Caucasian female who was diagnosed with LPD at the age of 24. Childhood was unremarkable for medical conditions, starting at the age of 17 she had recurrent episodes of prolonged abdominal pain with nausea and vomiting. During multiple admissions to the emergency department, abdominal X-rays showed repeatedly intestinal obstruction. No further investigation regarding the cause for this abdominal pain was undertaken until the age of 24. During an abdominal gynecological laparatomy -conducted because of recurrent abdominal pain and dysmenorrhoe- multiple polypoid pedunculated lesions on the large bowels were seen (see Figure 1), biopsy was not carried out because the lesions were well vascularized. On surgical abdominal exploratory laparotomy about 50 fibromatous tumors were found and removed from the serosal surfaces, small and large bowels, omentum and from the uterus. Histopathological analysis of these tumors revealed benign spindle cell tumors, classified as leiomyoma. Electron microscopy showed peculiar nodules consisting of mature fibroblasts, abundant collagen with a few admixed myofibroblasts. At the age of 29 the patient had another episode of severe abdominal pain, nausea and vomiting and abdominal distension, partial small bowel obstruction was seen again on abdominal X-rays. The patient had no further episode of abdominal discomfort until now and presented to us for further evaluation with abdominal bloating and discomfort. Other recorded medical conditions include Raynaud's disease (diagnosed at the age of 19), Prurigo nodularis with lichenification of the skin (onset of symptoms in adolescence, see Figure 2) and recently diagnosed postural orthostatic tachycardia syndrome (diagnosed at age 44).

Her clinical exam of the abdomen revealed nothing except abdominal distension, no masses or tenderness were noted. Inspection of the skin revealed dry lichenificated skin on all extremities with prominent itching nodules (see Figure 2). Laboratory findings were normal at admission.

The mother of the patient (III10) is now at age 82 and was diagnosed with Leiomyomatosis peritonealis disseminata at the age of 25. She also had recurrent episodes of severe prolonged abdominal pain and underwent exploratory laparotomy due to a ruptured appendix. Incidentally a large number of tumorous lesions was found, which were later on classified as leiomyomas. Interestingly this patient also was diagnosed with Raynaud's disease and has Prurigo nodularis-like skin lesions. The sister of this patient (III13) died at age 16 of peritonitis and sepsis. On post mortem examination intestinal obstruction due to multiple tumorous lesions was found as origin of peritonitis. Another brother (III17) died of peritonitis and gangrene of the bowels at the age of 20, resulting from intestinal obstruction of multiple pedunculated tumors (as reported by operating surgeon, 1939).

The grandfather (II3) of the propositus never was diagnosed LPD but has a past medical history of abdominal problems, including intestinal obstructions. Unfortunately no further information was available.

Three more members of the family are affected by LPD and furthermore have been diagnosed with Raynaud's disease and show Prurigo nodularis with skin lichenification. All three patients (IV2, IV3, IV4) had abdominal problems (e.g. prolonged severe abdominal pain) in their late teenage years and underwent explorative laparotomy, which showed more than 50 tumors in every case. The receptor status for progesteron- and estrogen-receptors was negative in these three patients. Patient V1 is the 10 year old son of patient IV2 and already had a complete intestinal obstruction, on explorative laparotomy more than 100 pedunculated tumors have been found in the abdominal cavity.

Patients IV5, IV7 and V2 reportedly have or had abdominal problems, but no confirmed diagnosis was available. No information on medical conditions, post mortem examinations or confirmed diagnoses was available for patients I1 and I2, grand-grandparents of the propositus (IV6).

## Discussion

Leiomyomatosis peritonealis disseminata (LPD) is a rare disease and only a relatively small number of cases have been reported so far. No epidemiologic data is available and cases reported have been from different ethnical groups, without any clear ethnical predominance. Except two cases, all cases describe female patients, so occurrence of LPD in males is extremely rare. No familial cases have been reported so far and no link to environmental influences has been found, except for excessive hormonal exposure [8].

Correct diagnosis is difficult and can only be achieved through invasive diagnostics together with histopathological analysis. Patients usually present with unspecific abdominal problems, e.g. abdominal discomfort or pain, rectal or vaginal bleeding (in cases with uterine leiomyomas) or symptoms of intestinal obstruction. Imaging studies revealed tumorous lesions in some cases [13], but in the propositus of this presented case no evidence of LPD could be found by any kind of imaging studies (ultrasound, computed tomography or magnetic resonance imaging). Biopsies can provide the material for a histopathologic diagnosis. However vascularized tumors can cause severe bleedings on biopsy, so that biopsies taken on explorative laparotomy seem to warrant greater safety and furthermore allow for assessing the extent of LPD or exclusion of other diagnoses. Differential diagnosis include peritoneal disseminated metastasis, malignancy or other forms of leiomyomas (e.g. benign metastasizing leiomyoma, a uterine leiomyoma associated with a smooth muscle mass in a solid organ). LPD is regarded as a benign disease, but in about 2–5% of the reported cases a progression towards malignancy has been reported [14-17]. Malignant transformation from leiomyoma to sarcoma was found on histopathological findings. However the majority of cases take a benign course, sometimes LPD resolves with hormone therapy or does not relapse after surgical resection. Some cases show recurrent growth of leiomyomas and recurrence of symptoms. The occurrence of LPD without intrauterine leiomyomas, in postmenopausal women [18], LPD with estrogen- and progesterone-receptor negative tumors [16] and the occurrence in men led to the idea, that LPD is a new disease entity [16,19].

**Figure 3:**
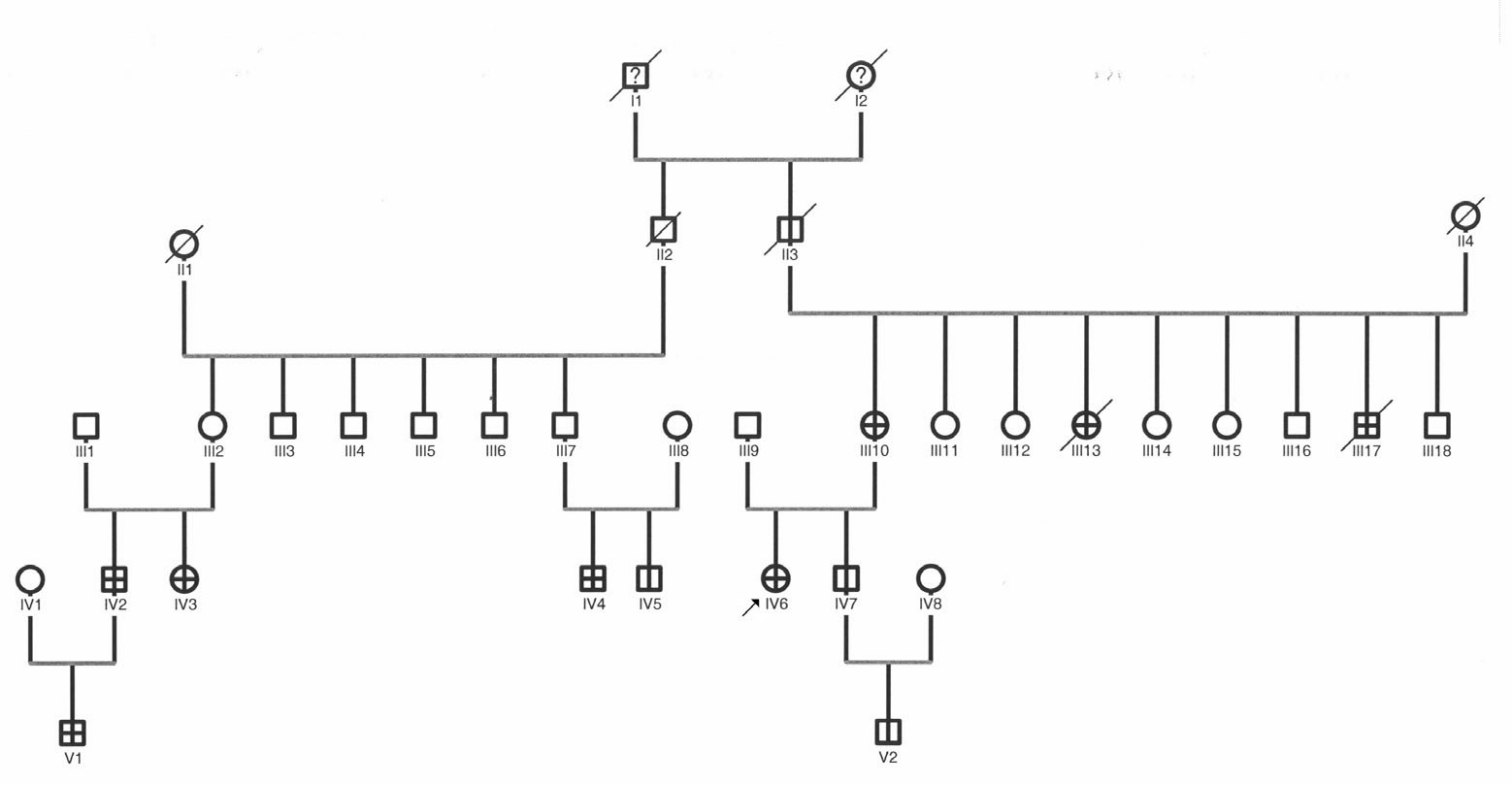
**Family pedigree. **Family pedigree (squares indicating males, circles indicating females, slashed symbols indicating deceased family members, single line indicates family members with abdominal symptoms but no diagnosis of LPD, double crossed lines indicate family members with diagnosis of LPD, question marks indicate family members without any medical information), additional diagnosis of Raynaud's disease and Prurigo nodularis is present in family members II10, IV2, IV3, IV4 and IV7.

In our reported case six siblings are affected by LPD, the two branches of the family links through the grand-grandfather of the propositus (IV6, see Figure 3, indicated by small arrow). There is no consanguinity and environmental influence is not very likely: different generations have been raised in different parts of the country and no other occurrence of LPD outside these families is reported. Assuming a genetic background of the familial clustering and taking the family pedigree into account we propose an autosomal-dominant model with varying degrees of penetrance. LPD appears in subsequent generations, skipping some generations and appears both in men and women. However other modes of inheritance cannot be excluded completely (e.g. genomic imprinting), but appear less likely. For an autosomal-recessive model a disease allele would have to be common in the population, requiring far more cases of LPD.

There is no known similar genetic disorder. However there are some similarities which can be taken into consideration. Mutations in the fumarase hydratase (FH) gene, an enzyme which is involved in the mitochondrial tri-carboxylic acid cycle, are known to cause leiomyomas [20,21]. Especially the dominantly inherited susceptibility to multiple cutaneus and uterine leiomyomas appears as hereditary leiomyomatosis. Furthermore, smooth muscle tumors have been linked to mutations in collagen genes [10]. Interestingly also skin lesions [22] have been found to be associated with collagen gene alterations. An alteration of a collagen gene as a common basis for familiar LPD seems possible. As a hypothesis, these two genes may be involved in the development of familiar LPD through unknown mutations. However we have no further data to verify these hypotheses.

## Conclusion

Leiomyomatosis peritonealis disseminata is a rare disease and familial clustering has never been reported and can be considered a unique appearance so far. Nevertheless, other cases may have never been diagnosed, because these leiomyomas can present only with minor symptoms or can go completely undetected and without symptoms. Leiomyomatosis peritonealis disseminata presents a hard to diagnose disease and is rarely considered as differential diagnosis.

This familial clustering of LPD should be genetically analyzed to find the underlying genetic pathomechanism. Only further diagnostics of all affected family members can reveal all aspects of this syndrome. Careful examination of all living family members, skin biopsies of all affected family members and a complete workup regarding Raynaud's disease could be future steps in a detailed investigation.

## Competing interests

The author(s) declare that they have no competing interests.

## Authors' contributions

All authors contributed equally to this work. All authors read and approved the final manuscript.

## Pre-publication history

The pre-publication history for this paper can be accessed here:
